# Setting the policy agenda for the treatment of substance use disorders in Iran

**DOI:** 10.1186/s12954-022-00612-w

**Published:** 2022-03-15

**Authors:** Saeid Mirzaei, Vahid Yazdi-Feyzabadi, Mohammad Hossein Mehrolhassani, Nouzar Nakhaee, Nadia Oroomiei

**Affiliations:** 1 Non Communicable Diseases Research Center, Bam University of Medical Sciences, Bam, Iran Sardaran Shahid Square- Shahid Rajaei Boulevard, 7616913555; 2 Health Services Management Research Center, Institute for Futures Studies in Health, Kerman University of Medical Sciences, Kerman, Iran; 3 Medical Informatics Research Center, Institute for Futures Studies in Health, Kerman University of Medical Sciences, Kerman, Iran

**Keywords:** Agenda-setting, Harm reduction, Policy analysis, Policymaking, Substance use disorder, Iran

## Abstract

**Background:**

Drug use is one of the most common public health problems globally. This study was done to analyze the agenda-setting of policies related to substance use disorder treatment in Iran since 1979.

**Methods:**

The current qualitative study was done through document review and interviews with policymakers and executives. Purposive sampling with snowball strategy was considered for sampling. Semi-structured interviews were done. A total of 22 documents were examined, and the data were saturated with 32 interviews. Kingdon's Multiple Streams Framework was used to analyze the data.

**Results:**

The results indicated the intersection of problem stream, policy stream, political stream, and opening the opportunity window. In the problem stream, the rapid growth of AIDS among people who inject drugs (PWID), the decrease in the average age of first drug use, the increase in the prevalence of substance use disorder in women, the ineffectiveness of compulsive treatment, and criminological perspectives played key roles. The policy stream included criminological perspective and war on drugs, and harm reduction. The political stream included announcing general anti-narcotics policies by the Supreme Leader of Iran and understanding the need for treatment, rehabilitation, harm reduction, and social support for substance use disorder by officials and policymakers.

**Conclusions:**

For a long time in Iran, policies based on the war on drugs were the dominant approach, and then, policies based on harm reduction and patient-centeredness were considered. The ideology and political parties influenced the executive apparatus's policy stream in this area. In countries with an ideological approach, the political stream plays a critical role in setting issues on the agenda. Therefore, policy entrepreneurs can put the points on the agenda by attracting the attention of political forces to the issue.

## Background

Nowadays, drug use and its unpleasant consequences are among the most common public health problems globally. Drug dependence has significantly increased in recent years and has become a humanitarian crisis, and the number of homeless people who use drugs is increasing day by day [1].

According to the 2019 report of the United Nations Office on Drugs and Crime, 35 million people worldwide suffer from substance use disorder. The number of people who inject drugs (PWID) globally is about 11.8 million, and the prevalence of Acquired Immune Deficiency Syndrome (AIDS) is 13.1%. Drug injections have been reported in 148 countries, with 120 reported Human Immunodeficiency Virus (HIV) infections in this population [2]. According to the 2019 report of UNAIDS, the number of people living with AIDS in the Middle East and North Africa (MENA) is estimated at 230,000. Also, 43% of new HIV infections were PWID [3]. Considering the harmful physical, psychological, cognitive, and social effects of the drug, pay attention to this issue [4].

For a long time, various approaches have been considered to solve this problem. For many years, the approach of combating the supply of the drug was the approach chosen by most countries in the world [5]. Most countries in the world welcomed this approach, and huge costs were allocated [6]. Over time, more studies were conducted on the causes of substance use disorder in the world. The findings of studies showed that substance use disorder is a multifaceted and complex phenomenon associated with different reasons. Therefore, this problem cannot be solved only by combating the supply of the drug. Despite the relentless efforts made in the field of compulsive and combating the supply of the drug, attention was paid to the field of demand. The demand includes several interventions to treat people who use drugs [7]. These interventions have been more or less welcomed by different countries and have spread widely globally [6], and Iran is not an exception. Iran is based in the MENA region [8]. Geographically, due to its proximity to Afghanistan and Pakistan (about 1800 km of the common border), the world's largest producers of opium and heroin, Iran is located in the east–west transit route and has a critical position [9]. Traditional drug traffickers use Iran's shortest possible route to transport the drugs produced in neighboring eastern countries from east to west. About 80% of the world's heroin is made in Afghanistan, and more than three-quarters of these drugs are trafficked through Iran and Pakistan [10]. Therefore, based on the strategic position of Iran, a significant threat is posed to the Iranian society [11, 12].

According to the global statistics, the prevalence of substance use disorder in Iran has increased from 1.61 in 1990 to 1.97 in 2017. Also, the rate of deaths due to drug use per 100,000 people in Iran has increased from 2.25 in 1990 to 3.68 in 2017 [2]. According to the 2018 statistics, there are 437,000 PWID in the MENA region, of whom about 200 000 are in Iran [13].

Like many other countries in the world, Iran has pursued the policy of war on drugs for many years. In 2004, at the initiative of the State Welfare Organization of Iran, the country moved toward harm reduction policy. In general, various treatment policies have been adopted during the past years regarding substance use disorder in Iran [14]. Although studies in substance use disorder have been conducted in Iran [15–17], the policymaking of drug abuse has received little attention. Therefore, the present study aimed to analyze the agenda-setting of policies related to treatment of substance use disorder in Iran since the beginning of the Iranian revolution in 1979.

## Methods

This study was conducted to identify the process of agenda-setting of policies related to treatment of substance use disorder in Iran since the beginning of the Iranian revolution in 1979 by reviewing documents and interviewing policymakers and executives. By documents, we mean all documents, policies, laws, and protocols related to substance use disorder in the areas of treatment and harm reduction in Iran, which were published from January 1979 to June 2021 and are available to the public. The research team purposively started identifying the key high-level documents through library search and referring to the websites of Expediency Discernment Council, Iran Drug Control Headquarters, General Directorate of Treatment and Social Support of Iran Drug Control Headquarters, Ministry of Health and Medical Education, Ministry of Cooperatives Labour and Social Welfare, State Welfare Organization of Iran, Islamic Parliament of Iran, and Social Council of Iran. The documents were selected based on Jupp quadruple considerations, including authenticity, credibility, representativeness, and meaning [18]. The research team set the original version of the documents is formally approved. National documents that were approved at the ministerial level and provincial level were reviewed. The content of each of these documents, which was related to the drug use and drug treatments, was selected for analysis.

A total of 22 documents were reviewed. The documents were studied separately by 3 members of the research team; some parts of the text related to the research topic were selected, and then, the content of these parts was analyzed. The reviewing documents were used to adapt and complete the data obtained from the interviews.

The research community of policymakers and executives includes all key informants who involved in the policymaking process of substance use disorder in the Ministry of Health and Medical Education, State Welfare Organization of Iran, Iran Drug Control Headquarters, State Prisons, and Security and Corrective Measures Organization, and Expediency Discernment Council at the national, provincial, and local levels. The research sample included key informative interviewees in the Ministry of Health and Medical Education, State Welfare Organization of Iran, Iran Drug Control Headquarters, State Prisons, and Security and Corrective Measures Organization, and Expediency Discernment Council and the affiliated groups that are directly involved in policymaking and executing policies related to the treatment of substance use disorder.

The sampling method was purposive with snowball strategy and maximum variation. The research team purposefully interviewed individuals involved in policymaking and implementation of drug policies according to the inclusion criteria. The inclusion criteria included at least 3 years of work experience in policymaking and executing policies related to the treatment of substance use disorder and harm reduction, informing interviewees about the policymaking and executing policies, having the most expertise in this area, and the willingness of the interviewees to participate in the research.

The research team then asked them to introduce other people who could have information in research (snowball strategy). The research team tried to have maximum diversity in the interviewees in that the interviewees were representative of different approaches and organizations to examine diverse approaches. The semi-structured interview and the interview guide were used to collect the data. After 32 interviews, the data were saturated. The distribution and characteristics of the interviewees are summarized in Tables 1 and 2, respectively. To analyze the qualitative data, the recorded interviews were accurately transcribed. Notes on the field and findings of the review of documents were added to this section. Kingdon's Multiple Streams Framework was used; in case of the emergence of new themes, they would be mentioned.

**Table 1:** Distribution of interviewees at three levels

Level	Position	Nos.
National	Department of Mental and Social Health and Addiction of Ministry of Health and Medical Education	1
	Expert of Mental and Social Health and Addiction of Ministry of Health and Medical Education	2
	Office of Prevention and Treatment of Drug Abuse, Department of Mental and Social Health and Addiction of Ministry of Health and Medical Education	1
	Member of the Treatment Committee of Iran Drug Control Headquarters	2
	Member of the Independent Drug Control Committee of Expediency Discernment Council	2
	Deputy for Addiction Prevention of State Welfare Organization of Iran	2
Provincial	Experts of the Addiction Office of the Welfare Organization of Kerman Province	2
	Deputy for Addiction Prevention of Welfare Organization of Kerman Province	1
	Addiction Specialists of Kerman University of Medical Sciences	2
	Department of Mental and Social Health and Addiction of Kerman University of Medical Sciences	2
	Supervision and Accreditation Management Unit of Kerman University of Medical Sciences	2
	Experts of Drug Abuse of Supervision and Accreditation Management Unit of Kerman University of Medical Sciences	2
	Department of Mental and Social Health and Addiction	1
	Secretariat of the Anti-narcotics Coordination Council of Kerman	1
	Therapist of the Anti-narcotics Coordination Council of Kerman	1
	Experts of the Anti-narcotics Coordination Council of Kerman	2
	Health and Treatment Department of Central Prison of Kerman	2
Local	Officials of methadone maintenance therapy centers, therapeutic community, drug courts, and drop-in centers in Kerman	4
Total	32

**Table 2:** Characteristics of interviewees

Education level	Frequency (%)	Gender	Frequency (%)
BA	6 (18.75)	Female	10 (31.25)
MA	2 (6.25)	Male	22 (68.75)
PhD/MD	24 (75)		

## Accuracy and robustness of data

To ensure the credibility of the data, the interviews and the extracted content were referred to the interviewees by the research team and their opinions were applied. Also, interviewing and reviewing documents were used to collect information for the credibility of the findings. Moreover, for ensuring dependability, two members of the research team were asked to extract the content of the interviews and did thematic analysis. Disputes on themes extracted by two researchers were discussed in a meeting with all members of the research team in order to reach a collective agreement on the disputed themes. Confirmability was achieved by preserving the documents in the qualitative stages of the research, as well as the prolonged engagement of the researchers with the research data and immersion in them. For ensuring transferability, the entire research process was recorded in full detail to follow the research path and external check.

## Results

In this section, the details of agenda-setting policies related to treatment of substance use disorder are examined and interpreted (Table 3).

**Table 3:** Summary of factors affecting agenda-setting of policies related to the treatment of substance use disorder

	Main class	Subclass
Agenda-setting	Problem stream	*Indicators:*
		Increase in drug detection and arrested people involved in drug crimes during the years after the Iranian revolution
		The rapid growth of the pattern of AIDS transmission among PWID
		Increase in high-risk sexual behavior among people who use drugs
		Decrease in the average age of first drug use
		Increase in the number of injecting and non-injecting people who use drugs
		Increase in prevalence of drug use in women
		Lack of effectiveness of compulsive treatment methods and criminological perspective
		Increase in the number of deaths due to drug use
		Increase in the social harms and crimes related to drug use
		*Focusing events:*
		AIDS epidemic among PWID in the prison of Kermanshah
		Announcement of general anti-narcotics policies by the Supreme Leader of Iran
		*Feedbacks:*
		Criticism to the health system due to the inability and unwillingness of the health system to treat substance use disorder
		Increase in people's demands and dissatisfaction of people with the government's performance in the field of substance use disorder
	Policy stream	Criminological perspective to drug use and the war on drugs
		Patient-centered perspective to people who use drugs and harm reduction
	Political stream	Announcement of general anti-narcotics policies of Iran
		Understanding the need for treatment, rehabilitation, harm reduction, and social support for drug dependent individuals by officials and policymakers
	Opportunity window	People involved in the health system of Iran (especially the State Welfare Organization and physicians)
		Scientific and academic groups
		Responsible international organizations

### Problem stream

The problem stream was influenced by indicators, focusing events, and feedback related to the issue, which attracted the attention of policymakers to the treatment of substance use disorder.

### Indicators

One of the cases indicating the unfavorable situation of substance use disorder in Iran was the statistics published. Statistics published by the Iran Drug Control Headquarters showed that from 1996 to 2006, the number of drug detection has more than doubled and continued to increase in subsequent years; this number was 620,000 kg for 2015. Also, the total number of people arrested for drug-related crimes increased from 139,000 in 1996 to 426,000 in 2006 [19]. The transmission of AIDS through sexual intercourse and injecting drug use in 1995 was 19 and 15 cases, respectively. In 2005, it was 175 and 195, respectively, indicating the rapid growth of the pattern of AIDS transmission among PWID. Reports show that the number of people living with AIDS among PWID was 24,000 in 2016, and 3800 people are added to this number every year [20].

This report showed that non-injecting drug users, who use less invasive and unsafe methods for drug use, did not have suitable conditions for HIV prevalence. On the other hand, it should be noted that reports indicated a significant prevalence of HIV among non-injecting drug users. There were also groups with high-risk sexual behaviors among drug users. In this regard, one of the interviewees stated: "Statistics and reports showed that the situation is terrible. The pattern of AIDS transmission and high-risk sexual behaviors in drug dependent individuals showed that the situation is not favorable …." (I. 3).

The research results in 2004 showed that the average age of people who use drugs was 21 years and the average age of the first drug use was 16.7 years [21]. Also, a rapid situational analysis study conducted by the State Welfare Organization in Iran in 2004 showed that 65.9% of people had started drug use under the age of 20 [19]. Since the low average age of the first drug use is one of the factors that can increase the prevalence of substance use disorder and the subsequent problems, this indicator was also influential in improving policymakers' attention.

Other critical indicators include the increase in the number of people who use drugs between 2000 and 2002; the number increased from 2 million to more than 3.5 million. The number of PWID increased by 7% during this period [22, 23]. The ratio of PWID to the total drug dependent individuals increased from 12.2 to 21.3% between 2004 and 2007 [22]. The high risk of drug use in PWID, in terms of overdose and disease transmission, attracted the attention of policymakers. Another indicator was the increase in the prevalence of substance use disorder among women in the society, which is referred to as the feminization of substance use disorder; according to the reports of the State Welfare Organization, the rate of women's drug dependent was 1% in the 1990s, 5% in the 2000s, and 5% to 10% in 2010 [24].

Other issues that significantly impacted changing the policy approach were the ineffectiveness of compulsive methods and criminological perspectives. Despite the widespread confrontation with the supply of drugs and the compulsive approach to substance use disorder, it was still one of the main challenges in Iran. For this reason, the need to change the approach to confronting drug use disorder was felt. But, that approach did not explicitly address those relevant goals of eradicating substance use disorder in Iran. In this regard, one of the interviewees stated: "We had a situation based on the encounters that we had in that period; not only did the number of people who use drugs reduce but also increased. According to the available data, more than 98% have returned to drug use, and we may not find the other 2%, or they might have died or withdrawn. At the same time, we said: Well, when this approach did not reduce the number of people who use drugs and even added to them, then, other things must be done…" (I. 11).

"It soon became clear that the concentration camps^*^^1^ did not work very well; not only those who were released from these camps became better, but also their substance use disorder worsened and became more greedy for drug use; the type of drug they used became more dangerous. … Another problem with these treatment centers was that these centers were not cost–benefit.” (I. 9).

Statistics of the Iranian Legal Medicine Organization also showed an increase in the number of deaths due to drug use between 2004 and 2007, so the death number rose from 4006 to 4713 [24]. On the other hand, substance use disorders played a significant role in many social harms; an interviewee said: "We focused on the costs posed to society. Many people may not know that many petty thefts that take place in the community are due to drug dependence. One may break the car glass to get money; s/he may break the car glass, pick up box wrenches, and sell it to buy the drug." (I. 1).

### Focusing events

Focusing events refer to those events and incidents that focus on problems. The AIDS epidemic among PWID was one such case.

The first case of HIV transmission through drug injection was observed in 1989, and by 1995, only about 5 to 10 new cases of HIV transmission were surveyed each year. However, with the outbreak in PWID in one of the prisons of Iran, Kermanshah, injecting drug use increased by 23 times in 1996 compared to the previous year, and it was mentioned, for the first time, as the most common way of HIV transmission. The number of cases of HIV transmission through injection by 2005 has been steadily increasing. In 2007, an average of 14.3% of PWID was HIV positive [24]. One of the interviewees said:

"One of the issues that helped to change the perspective and the policy was the AIDS epidemic in the prison of Kermanshah; all of a sudden, the number of HIV positive PWID increased by 20 times, so our policymakers should have stopped this issue, we needed to doing something …..”(I. 15).

With the increase in the prevalence of infection in this group, Iran entered from a low prevalence stage to a concentrated prevalence stage. Due to the association of the HIV epidemic with covert and stigmatized behavior in some marginalized members of society, namely PWID, the national response became very difficult. The epidemic was a landmark event, sounding the alarm and drawing public attention. Iran's location in the transit route of the drug, and especially its neighborhood with Afghanistan as the largest producer of opium in the world, increased the substance use disorder rate in Iran, especially the spread of injecting drug use and spread of HIV.

One of the events that strongly influenced the policy in this area was the announcement of general anti-narcotics policies by Iran's Supreme Leader. Before 2006, the Supreme Leader of Iran had verbally announced anti-narcotics policies to organizations. Still, in 2006, under the first paragraph of Article 110 of the Constitution, he announced the policies of this area in writing. Written communication of policies helped to align policies in this area. In this regard, one of the interviewees said:

Since 1994, the Iran Drug Control Headquarters realized the need for national policies in the field of drug use. Therefore, to implement the first paragraph of Article 110 of the Constitution, the Headquarters proposed to the Supreme Leader to integrate approaches in this field by determining a national program.

### Feedbacks

The feedbacks that highlighted substance use disorders are generally divided into two areas; criticism to the health system and increase in the people's demands, and public dissatisfaction with the government's performance in controlling substance use disorder.

One of the feedback provided to the health system that led to identifying the drug problem was its inability and unwillingness to solve problems and treat substance use disorder; at different periods, the health sector refused to perform its duties for various reasons. It has also been able to do so due to the lack of resources and facilities and was, therefore, criticized. In this regard, an interviewee stated:

"General policies say take people who use drugs and treat them. It is also supported by law, but its health system doesn’t accompany it. Access to treatment for people who use drugs is difficult, so there is a gap between general policy and executive policy. When the level of access to treatments such as methadone maintenance therapy is not high and timely, a series of people who use drugs come to the streets, demands rise and, then, they say to take them. We say that the health sector should take and support them. They say that I can’t, for whatever reason. The government is intervening here. The police take responsibility and deliver them to the drug courts very well. The health system is responsible for the intensification of judicial and disciplinary executive function…..."(I. 23).

The existence of drug dependent individuals in the city has caused a feeling of insecurity for the community, and it led to a public demand to solve this problem. Increasing demands of people also led to a better understanding of the policymaker and identifying the problem. On the other hand, according to opinion polls, unemployment, inflation, and drug users were named the people's main concerns. In this regard, the interviewees say:

“Substance use disorder has become a constant concern of people and has created a kind of distrust of the people, in a way that people inadvertently become dissatisfied with the performance of the government and say that the government either does not want or cannot deal with this phenomenon. Something had to be done …." (I. 12).

### Policy stream

Policy stream refers to solutions and policy options to solve issues and problems. In general, the policymaking of substance use disorder treatment can be divided into criminological perspective, compulsive treatment method, the war on drugs, and harm reduction.

In the early years of the Iranian revolution, due to fundamental changes in the philosophy of governance and problems of the new government and, then, due to the onset of the Iran-Iraq war, the dominant view of drug use was the war on drugs. The policies of that period included the maintenance and rehabilitation of people who use drugs in the camps and the approval of the National Program for Prevention, Treatment, and Rehabilitation of Drug Abuse in 1994, the opening of reception, follow-up, and treatment units for self-reported people who use drugs in the State Welfare Organization and membership in the self-help groups.

The standard approach of all these actions was the war on drugs and withdrawal. Considering the ineffectiveness of previous policies and the lack of reduction in the number of people who use drugs and treatment of people who use drugs, as well as the existence of local and international evidence of the effectiveness of treatment methods and the presence of evidence that indicated the existence of the problem stream, the criminological perspective gradually faded. The view of harm reduction emerged as a rival to the previous approach. In line with the harm reduction approach, various opioid substitution therapies and other drug dependence treatments such as opioid agonist maintenance treatment, Drop-in Centers (DICs) were established.

Needle and syringe programs, contraceptive methods, methadone, warm food and bathing, counseling services, and diagnostic tests, including HIV, are provided in DICs in Iran. Forming mobile response teams to provide services such as gathering of used needles and syringes, safe injection training to people who inject drugs, introducing the DICs and their benefits to homeless people who use drugs, distribution of condoms, sanitary napkin, needles and syringes to outreach groups are other DICs activities. There are few DICs for women in Iran and provide similar services, with the difference that due to the religious context of Iran, they provide services exclusively for women.

### Political stream

In the policies related to substance use disorder treatment, two issues had a significant impact on supporting the perspective of harm reduction and changing the approach, which will be discussed below.

The announcement of general anti-narcotics policies by Iran's Supreme Leader is one of the vital turning points in policymaking in this field. With the announcement of these policies, the discourse differences faded, and all proposed policies were aligned. On the other hand, the announcement of these policies reflected the supportive view of the government on the issue of harm reduction and treatment of people who use drugs. In this regard, one of the interviewees said:

"The approach of Supreme Leader of Iran on the substance use disorder is that this situation is not normal. … In fact, he has always had a problem with drug use but has never opposed treatment and harm reduction. For example, in paragraph 5 of the general anti-narcotics policies, he explicitly states that drug use must be criminalized. Still, he excludes treatment and harm reduction programs, which means that he supports this view." (I. 21).

On the other hand, the Supreme Leader of Iran in social harm management in the country proposed a national division plan to the Ministry of Interior and identified 5 priorities to solve the problem, among which were substance use disorders. He called on all country organizations to make an organized, scientific, and trans-organizational effort and a national coalition to prevent and deal with social harms and problems. The request of the Supreme Leader of Iran, as the highest position in the policymaking area of ​​the country, created an excellent opportunity for more cooperation of all relevant organizations to reduce the harm. On the other hand, the formation of the second wave of AIDS among PWID in the prison of Kermanshah and the risk of increasing the prevalence of this disease caused the officials to understand the need to address this issue more than before and to have a supportive perspective in this regard. Iran's accession to Single Convention on Narcotic Drugs in 1961 and amendment to the convention in 1972, Convention on Psychotropic Substances in 1971, political declaration guiding principles of drug demand reduction, and other resolutions of the 20th Special Session of the United Nations General Assembly in 1998 demonstrated the commitment of Iran and officials to reduce demand for drugs. In this regard, one of the interviewees said:

Thank God in the mid-2000s, the officials in charge in various sectors agreed that the issue of substance use disorder is critical and should be addressed, although it was addressed before, this time, it is different. International and national evidence confirmed that criminological perspectives were not responsive, and the views had become more scientific (I. 18).

## Discussion

At the beginning of the twentieth century, there was no international regulation on drugs. With the International Opium Commission formation in 1909 by the USA, the first multinational commission was formed. This meeting laid the foundation for a global ban on non-medical and scientific drug use [25]. Almost all countries ratified it. These countries were required to enact domestic laws that defined the production, manufacture, distribution, trade, use, and possession of drugs as criminal activities. It also required the countries to provide adequate treatment to people who use the drug and seek assistance [26]. In 1971, the USA introduced the concept of war on drugs [25]. Although this new approach was costly and did not reduce drug-related death, disease, or crime, it was copied by many countries [27]. In this approach, data on drug use were more important than drug use outcomes. Until 1981, the Centers for Disease Control and Prevention (CDC) announced the discovery of a new epidemic, later called AIDS [28].

Despite the growing number of overdose deaths, rising drug-related crimes, prisoner numbers, and government expenditures, there was an increasing reliance on the war on drugs approach. As the HIV crisis deepened, in 1984, the CDC stated that not sharing needles and drug injection equipment was effective in HIV transmission [26]. With growing evidence from the WHO in the last two decades of the twentieth century, in response to the vital role of people who inject drugs in the spread of HIV, drug policy has been increasingly influenced by the harm reduction concept [26]. In 1988, the first needle exchange program was launched in the USA. In 1995, the results showed harm reduction is a comprehensive HIV prevention strategy. In fact, the HIV/AIDS epidemic served as an opportunity window to put harm reduction policy on the international agenda-setting. This approach change has affected the drug policy of many countries, such as Iran [26, 28]**.**

### Opportunity window and policy entrepreneurs

The formulation of policies related to substance use disorder treatment in Iran is influenced by the occurrence of different currents in policymaking. Various indicators and reports related to the increase in the prevalence of drug use and subsequent results in Iran, negative feedback on the performance of the Ministry of Health and Medical Education, and increasing public demands intensified the problem and created the problem stream [22, 29]. In the problem stream, Kingdon cites changes in indicators, feedback, and focusing events as signs that inform groups and communities about issues. In his view, changes in statistical indicators can potentially indicate the occurrence of the problem. If this fluctuation is confirmed based on reliable data and scientific studies, it can focus on political decision-making [30].

On the other hand, the announcement of general anti-narcotics policies by the Supreme Leader of Iran and understanding the need for treatment, rehabilitation, and harm reduction by officials and policymakers provided the political stream to create the opportunity to accept the issue and the presence of harm reduction policies. Harm reduction policies were implemented as effective policies in different countries [31] such as Germany, the Netherlands [32], Canada [33], Australia [33] in comparison to war on drugs policies. However, there was no suitable platform for implementing this policy in Iran before this. The war on drugs, first proposed by US President Nixon, was dominant in some parts of the world, including Iran [34]. It should be noted that although the harm reduction approach has been set on the agenda, there is still an approach of war on drugs as a competing approach. Depending on the political party in the Presidency of Iran, one of the two approaches will be strengthened, and the other will be marginalized. Kingdon points out that the political streams refer to issues such as national morale, elections, government change, and the activities of interest groups. The political stream affects the political situation of society, which influences the choice of solutions [35].

According to Kingdon's Multiple Streams Framework, these multiple streams come together at a specific time and intersect. This is more likely to happen when an opportunity window opens. This window opens either by drawing attention to problems or creating political opportunities. In such a situation, a particular subject has the chance to become an option [30]. These streams provided vital opportunities for policy entrepreneurs to promote and pursue essential policy issues. According to Kingdon, policy entrepreneurs are organizers and catalysts of change that help agenda-setting, policy development, and implementation [35]. As mentioned, with the problems raised in the issue of drug use, especially in the form of credible reports with statistics, policy entrepreneurs have become more sensitive. They have provided more ideas to solve the problem.

Policy entrepreneurs in substance use disorder treatment were specifically active on 3 fronts and shaped the policy stream. These entrepreneurs included people related to the health system, especially the State Welfare Organization and the Ministry of Health and Medical Education, especially physicians, scientific and academic groups, and international organizations. The health system sought to expand prevention and treatment activities. The scientific and educational groups presented international policies and evidence as ideas, and responsible international organizations showed the dimensions of the problem and the solution. However, according to Kingdon's theory, no policy is formed as long as there is no political stream. As before, substance use disorder treatment and harm reduction policies were implemented cross-sectionally and sporadically in some parts of Iran but generally were not set on the agenda. Therefore, as mentioned, with the announcement of general anti-narcotics policies by the Supreme Leader of Iran, policies of substance use disorder treatment were formed, and policy entrepreneurs linked 3 streams and set the issue on the agenda [36]. Findings of an investigation by Watson et al. showed that physicians' support has been influential in the success of harm reduction policies [37]. The State Welfare Organization, especially the Deputy for Prevention and the head of the Addiction Office of the mentioned organization, played an active role in creating ideas and agenda-setting. They have been the first policy entrepreneurs to provide evidence and information to shape the policy stream since 1996, and the efforts paid off in 2006 with the formation of the political stream. On the other hand, the number of medical graduates increased in the 2000s, and a large number of physicians became interested in substance use disorder treatment. The group's community support was also effective in the agenda-setting of substance use disorder treatment.

## Conclusion

Multiple streams show their sensitivity to different dynamics in problem definition, solution formulation, and political processes that bring these streams together at the right time. The study's findings showed how each indicator, feedback, and the focusing event were effective in raising the issue. Although the policy stream helped to highlight the issue and increase the attention of policymakers, the political stream played a significant role in the announcement of general anti-narcotics policies by the Supreme Leader of Iran, as a strong political force helped the agenda-setting of this issue. The political stream has shown war on drugs and harm reduction approaches. For a long time, the war on drugs policy was the dominant approach and, then, harm reduction and patient-centered policies emerged as rivals for the previous approach.

Meanwhile, the policy stream in this area was influenced by the ideology and political parties in the executive apparatus. In general, in countries with an ideological approach, the political stream plays a critical role in setting issues on the agenda. Therefore, policy entrepreneurs can pave the way for putting the issue on the agenda by attracting the attention of political forces to the issue through various means such as mass media, scientific studies, and statistics.

## Data Availability

The datasets used and analyzed during the current study are available from the corresponding author on reasonable request.

## Footnotes

1 * camps where notorious people who use drugs are forcibly sent by order of a judge and are not given any drug to withdraw.
